# Case report: Impact of mixed reality on anatomical understanding and surgical planning in a complex fourth ventricular tumor extending to the lamina quadrigemina

**DOI:** 10.3389/fsurg.2023.1227473

**Published:** 2023-08-22

**Authors:** Elisa Colombo, Delal Bektas, Luca Regli, Tristan van Doormaal

**Affiliations:** Department of Neurosurgery and Clinical Neurocenter, University Hospital Zurich, Zurich, Switzerland

**Keywords:** mixed reality, 3D visualization, fourth ventricle, tumors of the IV ventricle, posterior fossa microneurosurgery

## Abstract

**Background and importance:**

Tumors of the fourth ventricle account for 1%–5% of all intracranial neoplastic lesions and present with different configurations and anatomical challenges. Microsurgery represents the primary therapeutic strategy for the majority of fourth ventricular tumors, and adequate anatomical understanding and visualization are paramount to surgical planning and success. The authors present the case of a young patient with a complex fourth ventricular tumor, whose surgery was successfully planned using a novel mixed reality (MxR) system.

**Case description:**

We present a case of a 31-year-old woman with a lesion extending from the fourth ventricle to the lamina quadrigemina and causing symptomatic hydrocephalus occlusus. Through the combined use of routine 2D images and an interactive 3D anatomical model, an interhemispheric transtentorial approach was used to remove 98% of the lesion with successful functional outcomes.

**Conclusions:**

The application of advanced 3D visualization with a novel MxR system to the surgical planning of a complex fourth ventricular lesion proved relevant in designing the best surgical approach and trajectory to better identify potential intraoperative challenges and rehearse the patient-specific anatomy. The present case report endorses the implementation of advanced 3D visualization in routine perioperative practice.

## Introduction

To date, the primary therapeutic strategy for tumors of the fourth ventricle is microsurgical removal (1–3). Lesions of the fourth ventricle may present with a wide range of anatomical variations and are in close proximity with extremely eloquent structures in the context of the anatomical complexity of the posterior fossa. Therefore, surgical planning is fundamental to tailor the microsurgical approach for complex fourth ventricular lesions, and it may not be optimal if planned based on two-dimensional (2D) images only. The application of three-dimensional (3D) visualization using mixed reality (MxR) may offer a better anatomical understanding, and it has an emerging application in neurosurgery (4). Furthermore, diverse MxR systems have been developed, and initial experience on their implementation in the perioperative phase has been gathered (5–7). The advantages of patient-specific interactive 3D anatomical models may be multiple and range over the design of the optimal surgical approach and intraoperative corridor, a realistic and comprehensive understanding of the close anatomical structures, and the pre-operative rehearsal and anticipation of the intraoperative challenges (8, 9).

The authors describe a successful surgical approach for a complex fourth ventricular tumor extending until the lamina quadrigemina and infiltrating the basal veins designed using a novel mixed reality system.

## Case description

**Figure 1:**
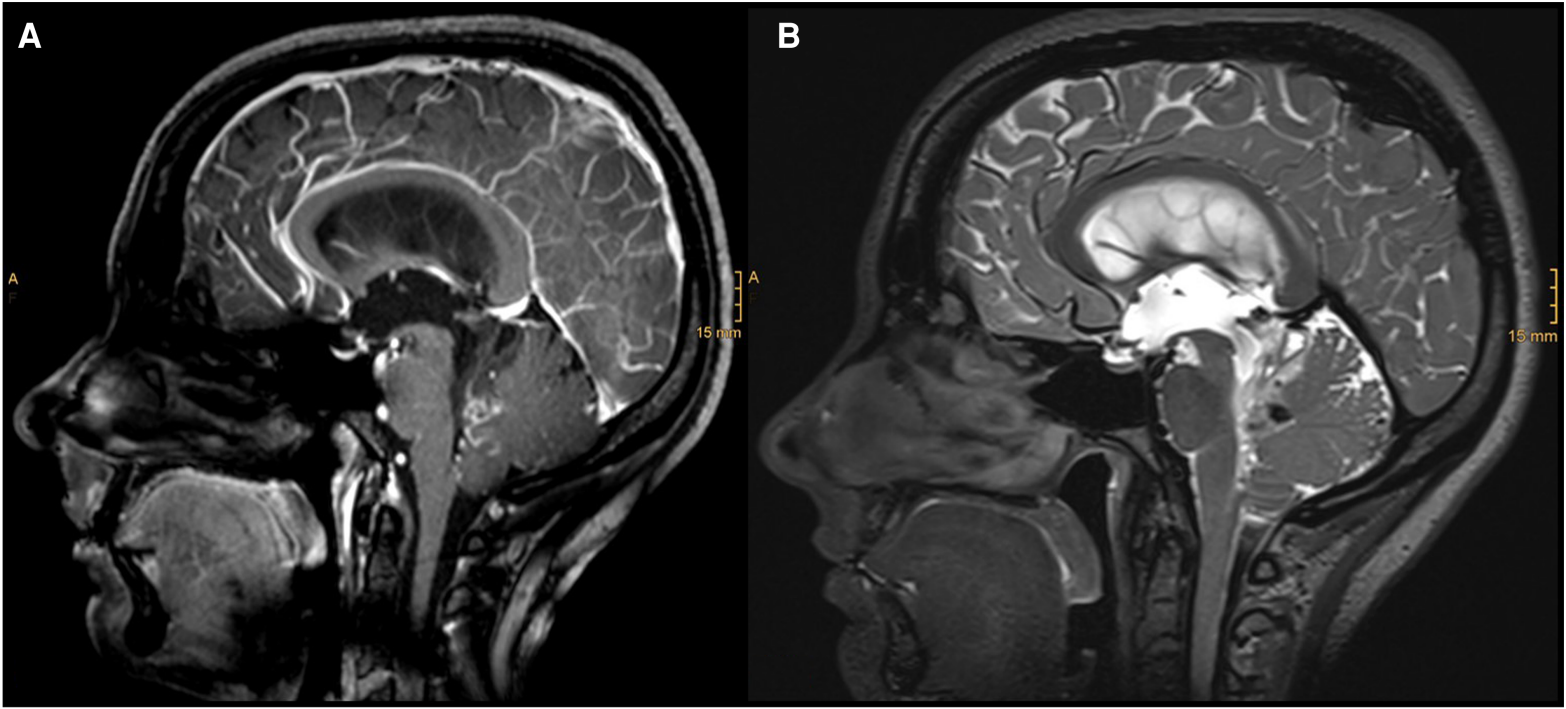
Diagnostic MRI study documenting a partially contrasted lesion of the IV ventricle with cystic components and infiltration of the vermis: (**A**) sagittal T1 with contrast; (**B**) sagittal T2.

A 31-year-old woman presented with a 5-day history of double vision in all directions, holocephalic headaches, and nausea. Radiologic workup showed a hyperintense lesion extending from the fourth ventricle to the lamina quadrigemina and causing a hydrocephalus occlusus (Figure 1). An external ventricular drainage (EVD) was immediately inserted, and 1 day after, the resection of the ventricular mass was scheduled.

Surgical strategy and patient positioning were discussed based on routinely used 2D DICOM (Digital Imaging and Communications in Medicine) images and further analyzed using 3D MxR technology.

### Mixed reality system

**Figure 2:**
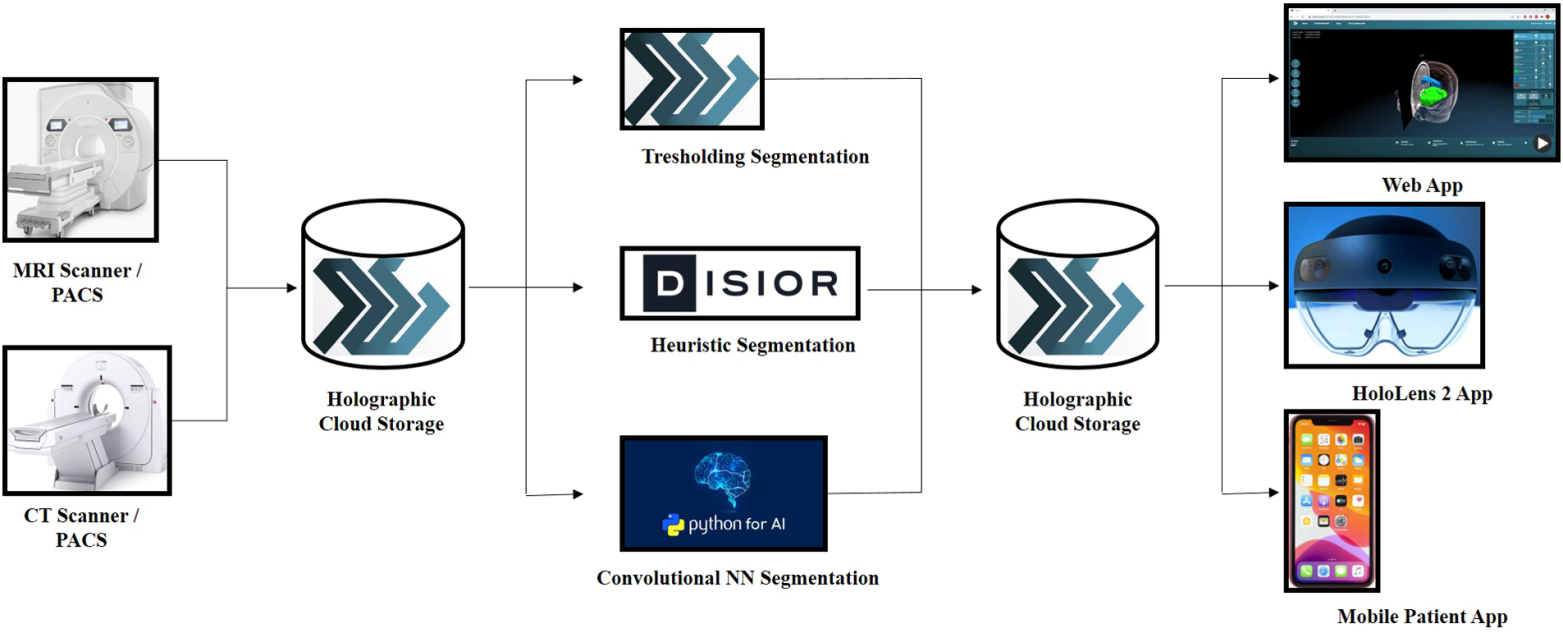
Schematic overview of the MxR system in all its components, starting from the imaging source, passing through the cloud-based storage and the strategies used for holographic rendering and ending with the visualization methods of the MxR output.

The novel MxR system utilized for holographic rendering and 3D visualization consisted of different components (Figure 2). The primary component was a CE-certified cloud environment (Lumi, Augmedit bv, Naarden, Netherlands) providing a direct MxR output using a validated expanding mesh algorithm (Disior, Helsinki, Finland) (10, 11). The Lumi cloud environment had associated applications for MxR glasses, which represented the second component of the system and the main tool for 3D visualization of the hologram (Hololens 2, Microsoft Corporation, Redmond, WA, USA).

### Hologram rendering

2D DICOM images from an external institution were loaded in a completely anonymized form on the novel CE-certified cloud environment (Lumi by Augmedit bv, Utrecht, Netherlands) starting from the acquisition of an axial T1 MPRAGE with contrast medium (Siemens Skyra 3 T MRI, TR = 1,800 ms, 168 slices, slice thickness = 1.0 mm, acquisition matrix = 256 × 232, FOV: 90.62, pixel spacing: 0.48828125). The magnetic resonance study was automatically segmented on the platform to highlight the skin, skull, brain, ventricular system, and tumor. Following the automatic segmentation, the tumor was further refined by means of manual segmentation on 3D Slicer (3D Slicer image computing platform | 3D Slicer) (12).

### Hologram interactive 3D visualization

**Figure 3:**
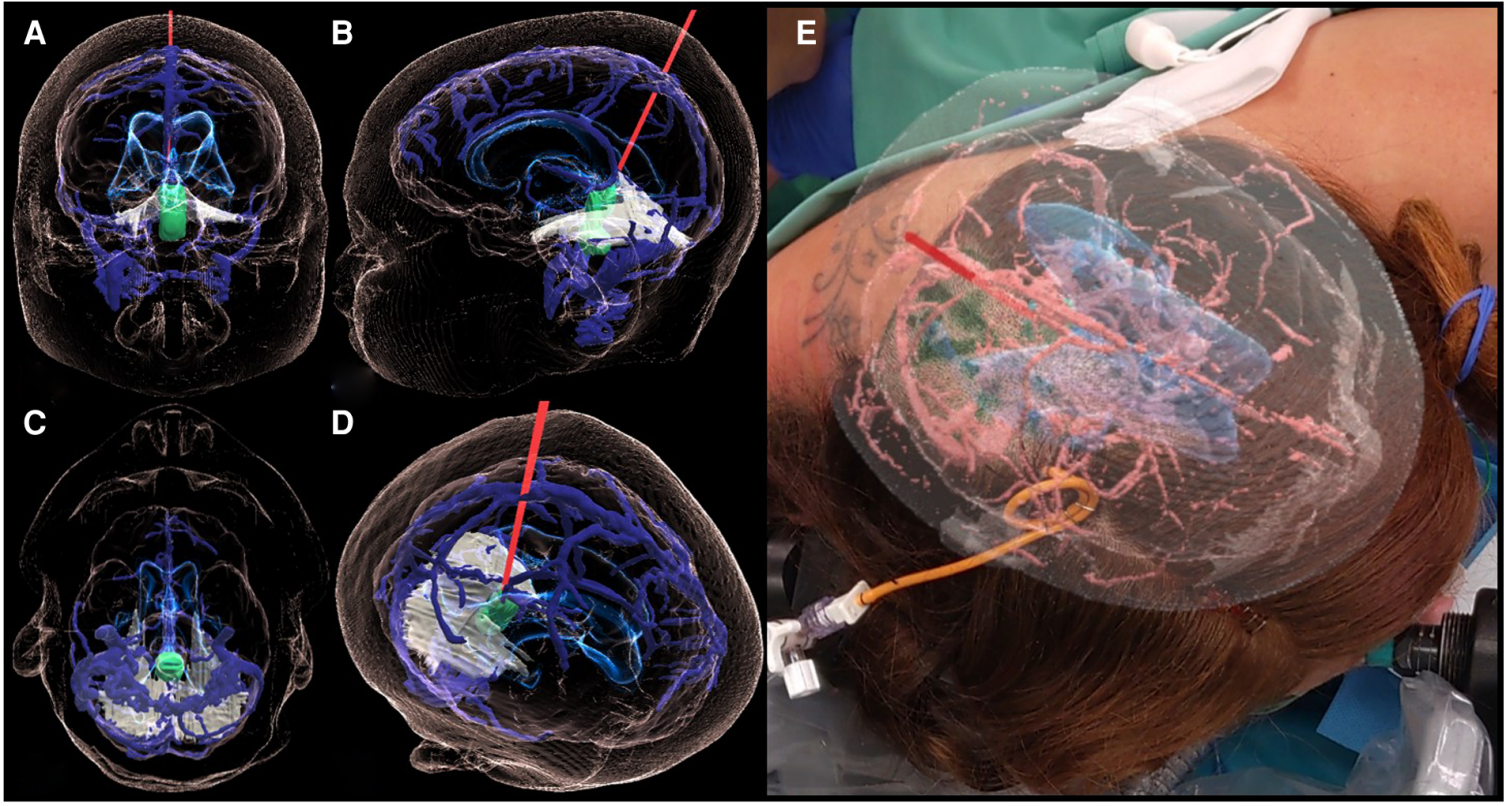
3D holographic rendering of the IV-ventricular lesion (green) and the intraoperative trajectory (red line) on the coronal (**A**), sagittal (**B**), axial (**C**), and a free (**D**) plane. Case report-specific application of MxR in the intraoperative environment showing the view of the operator wearing the MxR glasses and interacting with the hologram while matching it with the patient's head (**E**).

Through the MxR glasses, the primary user could visualize the hologram in 3D and interact with it and project it onto the real anatomy of the patient, manually matching it using her head to review and plan surgical positioning and the approach (Figure 3).

### Surgical strategy

To manage the increased intracranial pressure caused by an occluded hydrocephalus due to the mass effect, a right frontal EVD was inserted a day prior to the removal of the tumor.

**Figure 4:**
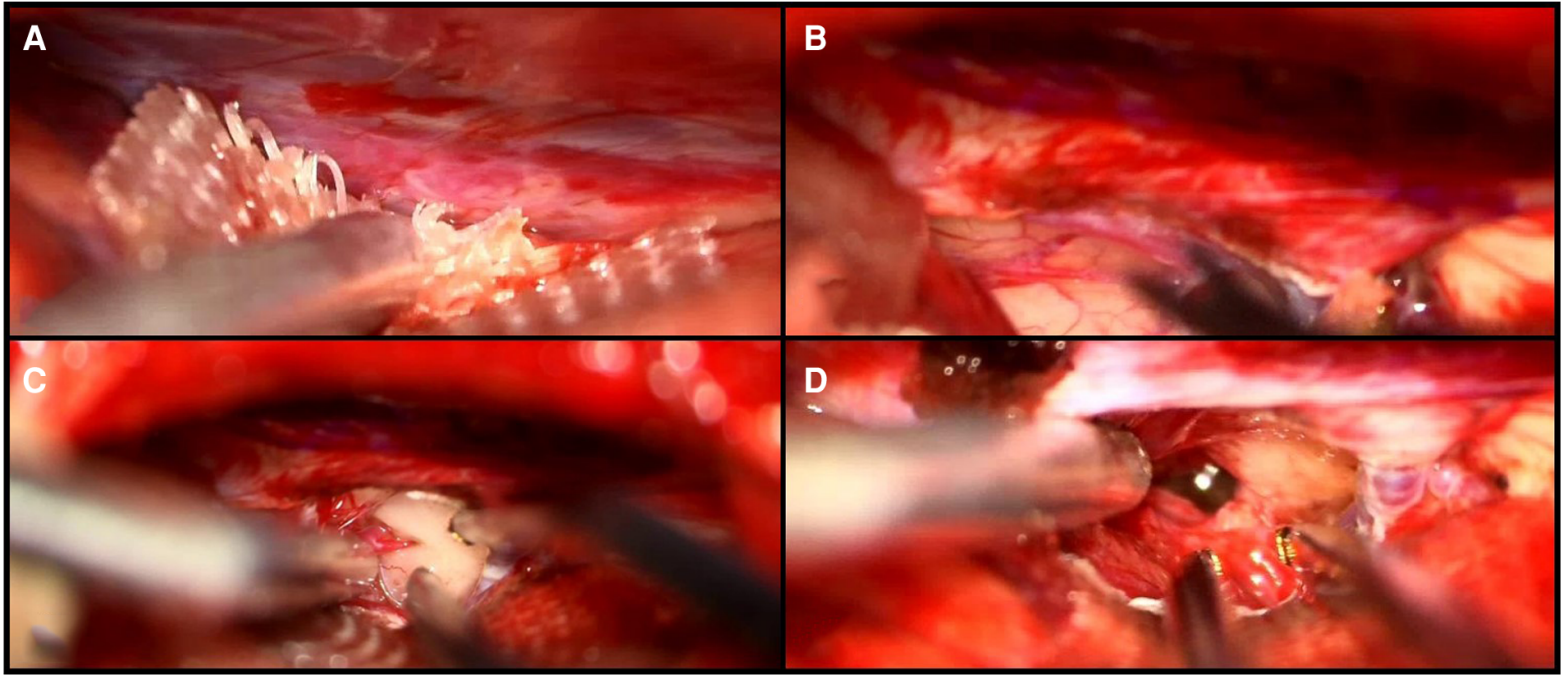
Intraoperative visualization of the falcotentorial margin (**A**), the small opening of the tentorium (**B**), the presentation of the tumor (**C**), and the IV ventricle after resection of the lesion (**D**).

Based on the combined analysis of 2D images and, most importantly, the 3D model, the patient underwent a parietal craniotomy with a subsequent interhemispheric transtentorial approach to the fourth ventricle. The MxR-3D visualization of the venous anatomy and the relationship of the tumor with the ventricular system was decisive in choosing a right parietal craniotomy to release maximally the right parietal vein frontally. This approach granted a straight trajectory posteriorly from the right parietal vein and right in the axis of the tumor. The 3D holographic visualization greatly helped in planning and rehearsing a surgical trajectory that could avoid the risk of injury of the basal cerebral veins. Furthermore, surgical planning with the 3D hologram endorsed the need to open the tentorium parallel to the straight sinus to achieve better intraoperative vision and control of the tumor (Figure 4). Patient positioning was designed according to the MxR planning, and the patient was positioned three-quarters prone with the head secured in a skull clamp slightly elevated and tilted toward the floor. A linear occipito–parietal incision along the midline was the approach chosen.

To confirm gross–total resection of the tumor, an intraoperative MRI was performed, revealing 98% removal of the lesion with a small remnant at the tectal level, which was too adherent to the great vein of Galen to be safely removed.

## Discussion

Fourth ventricular tumors account for 1%–5% of all intracranial neoplastic lesions and represent two-third of the lesions of the ventricular system (1, 2). To date, the classical and oldest approach to removing tumors of the fourth ventricle is the median suboccipital approach, which allows access to lesions of the cerebellar hemispheres, vermis, and medulla (1, 13). This approach is beneficial for extended anatomical exposure, enabling good surgical orientation and intraoperative manipulation. However, it is potentially associated with more tissue injury, the risk of sinus damage, and postoperative cerebrospinal fluid leakage (14). Another relevant surgical approach to lesions of the fourth ventricle is the telovelar approach, which exploits the natural space of the cerebellomedullary fissure to expose the tela choroidea and the inferior medullary velum (14, 15). The telovelar approach provides good access to the lateral recess, but exposing the posterolateral and the superolateral recesses is challenging and may require the removal of the cerebellar tonsils (14). The median suboccipital and telovelar approaches may be performed in a standard fashion or slightly modified depending on the anatomical presentation and extension of a fourth ventricular tumor. In order to tailor the surgical approach in a lesion-specific manner, an accurate pre-operative study of the anatomy of the lesions and their relationship with the surrounding structures is fundamental. In this regard, implementing augmented reality and modern 3D visualization techniques in neurosurgery has been qualitatively beneficial (16). Furthermore, applying MxR to surgical planning of complex cases gives the benefit of a non-threatening environment for less experienced surgeons, who can rehearse the procedure, foresee intraoperative difficulties, and increase surgical confidence (4).

The authors reported a complex case for which the use of MxR with an interactive patient-specific 3D anatomical model in adjunct with routine 2D images proved advantageous in gathering a better anatomical understanding and customizing the surgical approach and the intraoperative trajectory. Furthermore, surgical planning with MxR allowed a distinct patient positioning and a more realistic pre-operative analysis of the intraoperative surgical risks and challenges.

## Conclusion

The application of advanced 3D visualization with a novel MxR system to the surgical planning of a complex fourth ventricular lesion proved relevant to designing the best surgical approach and trajectory to better identify potential intraoperative challenges and rehearse the patient-specific anatomy. The present case report endorses the implementation of advanced 3D visualization in routine perioperative practice.

## Data Availability

The original contributions presented in the study are included in the article, further inquiries can be directed to the corresponding author.

## Supplementary material

SUPPLEMENTARY FIGURE 1

Authors provided the possibility to visualize the hologram with a smartphone by scanning the QRCode provided below. The hologram is protected by a PIN: 715-899.
