# Supplementary figures and images for: Case report: Impact of mixed reality on anatomical understanding and surgical planning in a complex fourth ventricular tumor extending to the lamina quadrigemina
Source: Front Surg. 2023 Aug 22;10:1227473. doi: 10.3389/fsurg.2023.1227473 (PMC10477590; doi:10.3389/fsurg.2023.1227473)

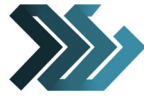

# AUGMEDIT

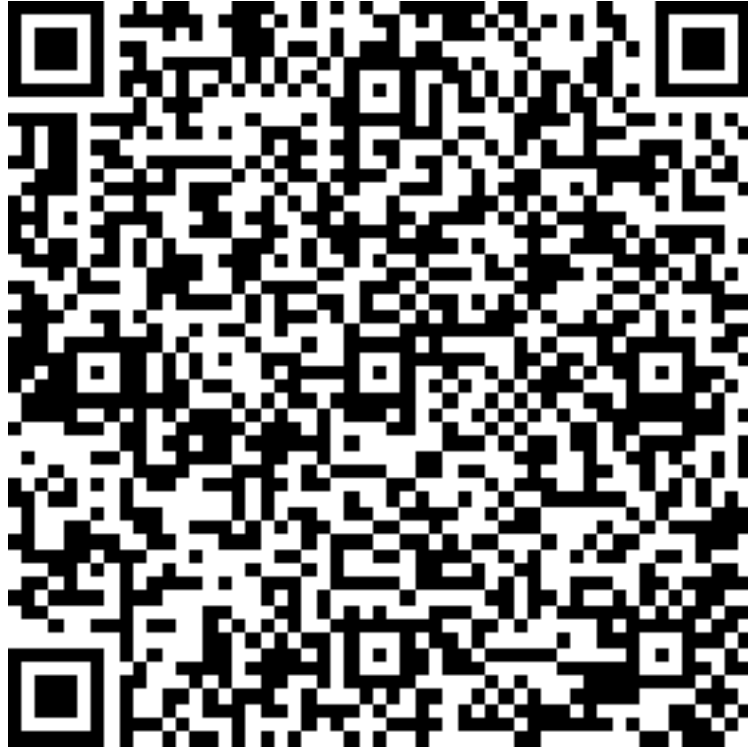

Scan QR with mobile device to see hologram

Supplement: Supplementary file 1 [file Image1.pdf]
